# Retraction Note to: The *BRCA2* variant c.68–7 T > A is associated with breast cancer

**DOI:** 10.1186/s13053-018-0093-1

**Published:** 2018-05-02

**Authors:** Pål Møller, Eivind Hovig

**Affiliations:** 1 Department of Medical Genetics, Research Group Inherited Cancer, Oslo University Hospital, Oslo, Norway; 2 Department of Tumor Biology, Institute for Cancer Research, Oslo University Hospital, Oslo, Norway; 3 Center for Hereditary Tumors, HELIOS-Klinikum Wuppertal, University of Witten-Herdecke, Wuppertal, Germany; 4 Institute for Cancer Genetics and Informatics, The Norwegian Radium Hospital, Oslo University Hospital, Oslo, Norway; 5 Department of Informatics, University of Oslo, Oslo, Norway

## Retraction

This article [1] has been retracted at the request of the authors. Upon re-review of the data, the authors identified coding errors in this study. Due to an error in the SQL query, the conclusions drawn in the article are incorrect. A re-examination of the data shows that there is no association between familial breast cancer and the BRCA2 variant c.68–7 T > A. Another recent study suggests that the variant is not pathogenic [2]. All authors agree to this retraction.
